# Supplementary material for: Experimental evolution of metabolism under nutrient restriction: enhanced amino acid catabolism and a key role of branched-chain amino acids
Source: Evol Lett. 2023 May 18;7(4):273–84. doi: 10.1093/evlett/qrad018 (PMC10355184; doi:10.1093/evlett/qrad018)
Supplement: qrad018_suppl_Supplementary_Material [file qrad018_suppl_supplementary_material.pdf]

Experimental evolution of metabolism under nutrient restriction: enhanced amino acid catabolism and a key role of branched-chain amino acids

Fanny Cavigliasso[1,4], Loriane Savary[1], Jorge E. Spangenberg[2], Hector Gallart-Ayala[3], Julijana Ivanisevic[3], Tadeusz J. Kawecki[1,5]

[1] Department of Ecology and Evolution, University of Lausanne, Lausanne, Switzerland

[2] Institute of Earth Surface Dynamics, University of Lausanne, Lausanne, Switzerland

[3] Metabolomics Unit, Faculty of Biology and Medicine, University of Lausanne, Lausanne, Switzerland

[4] E-mail: *fanny.cavigliasso@gmail.com*

[5] E-mail: *tadeusz.kawecki@unil.ch*

Electronic Supplementary Material

Detailed Methods 2

Supplementary Discussion 11

Supplementary Tables S1-S9 (S3 is a separate file) 14

Supplementary Figures S1-S6 (S1 is a separate file) 18

Supplementary Data separate files (“Raw_data_metabolomics”, “Raw_data_BCAA and WKK supplementation”, “Raw_data_15N enrichment”, “script metabolomics BCAA_WHK 15N_enrichment”)

Detailed Methods

Experimental evolution

We used six Control and six Selected populations of *Drosophila melanogaster* originally derived from a sample of wild flies collected in Basel, Switzerland, in 1999 and adapted to the lab conditions over five years prior to the commencement of the evolution experiment. Details of the experimental evolution protocol are described in Kolss *et al.* (2009). Briefly, the Control populations have been maintained on 3-week generation cycle on a standard larval diet medium (15 g agar, 30 g sucrose, 60 g glucose, 12.5 g dry yeast, 50 g cornmeal, 0.5 g $CaCl_2$, 0.5 g $MgSO_4$, 10 mL Nipagin 10%, 6 mL propionic acid, 20 mL ethanol per liter of water), at a controlled density of approximately 200-250 eggs on 40 mL of food. The six Selected populations have been maintained with the same generation cycle and larval density on a poor larval diet containing one-fourth of sugars, yeast and cornmeal content present in the standard diet. After their emergence, adult flies of both regimes were transferred to standard diet and additionally fed with live yeast *ad libitum* before egg collection. The census population size was about 200 reproducing adults. Flies were reared at 25°C with 50–70% humidity and 12:12 light cycle. The experiments described in this paper were performed after 244 to 287 generations of experimental evolution.

Before each experiment, we reared the 12 populations for at least two generations on standard diet to minimize environmental maternal effects. To obtain larvae for each experiment, we let approximately 200 adult flies from each population lay eggs overnight on orange juice-agar plates supplemented with yeast. The desired number of eggs was then transferred onto the experimental media (see below) and inoculated with feces suspension from a pool of adult flies from all populations to ensure homogeneity of larval microbiota.

To prepare the feces inoculum we haphazardly collected 10-15 flies per population and transferred them into a petri dish containing a small slice of standard diet medium. After 48 h we removed the flies and the medium slice from the petri dish, washed the petri dish with PBS using a brush, filtered the resulting feces suspension through a fine mesh to remove eggs and debris,

and adjusted the suspension to OD_{600} = 0.5 with PBS. Each larval culture bottle was inoculated with 300 µL of this inoculum prepared on the same morning.

High-coverage targeted metabolomics

We performed high-coverage or multiple pathway targeted metabolomics, targeting several hundred metabolites involved in glycolysis, pentose phosphate pathway, purine and pyrimidine metabolism, TCA cycle, amino acid metabolism and oxidative phosphorylation. We used a factorial design whereby larvae from all Selected and Control populations were assayed on both diets, with three replicate samples per population and diet, in three batches per diet (one sample per batch and population). Due to workload samples from different diets were collected on different dates.

Sampling

Larvae from all populations were raised on either diet at the density of approximately 200 eggs per bottle with 40 mL of food. In late third instar larvae a pulse of ecdysone is produced to initiate metamorphosis, which can have a large impact on metabolism and generate artifacts in the analysis (Lavrynenko *et al.* 2015; Li and Tennessen 2018; Kannangara *et al.* 2021). We aimed to study larval metabolism during growth phase before these pre-metamorphosis changes take place, in early third instar larvae. To assure a relative uniformity of their stage despite large differences in the rate of development due to diet and adaptation to it, we first transferred late second instar larvae (identified by "clubbed" anterior spiracles, http://www.shingletonlab.org/instars) from poor or standard diet to another dish of the same diet. Approximately 15 h later we collected samples of 15-20 larvae that transitioned to the third instar (recognizable by the presence of "branched" anterior spiracles). Using multiple larvae per sample provided sufficient material for the analysis but also reduced sampling variation due to sex, precise stage, genotype and developmental history of individual larvae. Following

recommendations (Cox *et al.* 2017; Li and Tennessen 2018), larval samples were washed three times in 200 µL of PBS, then centrifuged at 2000 g and 4°C for 1 min to pellet larvae and remove any extra liquid that might interfere with mass spectrometry measurement. The pellets were snap-frozen and stored at -80°C.

Multiple Pathway Targeted Analysis

The samples were analyzed by Hydrophilic Interaction Liquid Chromatography coupled to tandem mass spectrometry (HILIC - MS/MS) in both positive and negative ionization modes using a 6495 triple quadrupole system (QqQ) interfaced with 1290 UHPLC system (Agilent Technologies). Data were acquired using two complementary chromatographic separations in dynamic multiple reaction monitoring mode (dMRM) as previously described (van der Velpen *et al.* 2019; Medina *et al.* 2020). Data were processed using MassHunter Quantitative Analysis (for QqQ, version B.07.01/Build 7.1.524.0, Agilent Technologies). Pooled QC samples were used to correct for signal intensity drift; metabolites with CV > 30% were discarded (Dunn *et al.* 2011; Tsugawa *et al.* 2014; Broadhurst *et al.* 2018). Linearity of metabolite response was verified with serially diluted quality controls (dQC); peaks for which the correlation with dilution factor was poor ($R^2 < 0.7$) were filtered out; resulting in 174 retained metabolites.

Data transformation and outlier removal

The raw values of peak areas were $\log_2$ transformed, zero-centered and Pareto-scaled (i.e. divided by the square root of the standard error). Then, we identified outliers with a robust PCA on the covariance matrix of the data (Hubert *et al.* 2005). We used the R function *PcaHubert* with the number of principal components set to 2. Based on a visual inspection of the diagnostic plot, six outlier samples have been identified and removed (corresponding to three samples with a score distance above the cutoff and three samples with an orthogonal distance above 15; corresponding to four Selected samples, one from standard diet and three from poor diet and

two Control samples reared on standard diet) (Supplementary Figure S6) (Hubert *et al.* 2005). We then fitted a linear mixed model for each metabolite, with the selection regime, the diet and their interaction as fixed factors and the replicate population, its interaction with diet and the batch as random factors. We then used *compute_redres* R function with "std cond" type to obtain Studentized residuals of each metabolite and sample. Thirty-two data metabolite-specific data points (belonging to 29 metabolites and 12 samples, 0.28% of all data point) with an absolute Studentized residual higher than 3.46 (likelihood of 0.001) were considered as outliers and removed. This last data set (i.e., 65 samples with 11278 data points) was used in the analyses described below (the data were not rescaled after outlier removal).

Multivariate analysis

To evaluate whether larvae reared on two different diets and from different selection regimes can be differentiated based on their overall metabolome, we performed a principal component analysis (PCA). using covariance matrix on log-transformed and Pareto-scaled concentrations (R package mixOmics (Rohart *et al.* 2017)). To avoid conflating two levels of replication (population and sample) and to reduce noise due to batch effects, we first obtained a single estimate of each metabolite's abundance per population and diet. To do so, we fitted a linear mixed model to the abundance of each metabolite (log-transformed and Pareto-scaled), with the population, the diet and their interaction as fixed factors and the batch as random factor. The estimated marginal means for each population × diet combination were obtained from this model (using R package emmeans) and used as input for the PCA and clustering heat map. To test whether the diet and evolutionary regime affect the position in the PC space, we performed a MANOVA on scores obtained from the first three PCAs as response variables, selection regime, diet and their interaction as fixed factors. We also performed clustering analysis using *heatmap* R function on the same data as the PCA.

Univariate analysis

To identify metabolites that differ between selection regime and diet, we fitted a linear mixed model (LMM) with Type 3 F-tests for each metabolite with *lmer* R function (Kuznetsova *et al.* 2017) and the same set of variables as in previous section. We then performed a contrast analysis in which we compared Control and Selected populations for each diet with *emmeans* and *pairs* functions in R. The p-values were corrected for multiple comparison using Storey q-value approach (pFDR; Storey and Tibshirani 2003). To help interpretation, we back-scaled contrast estimates towards $\log_2$ fold change (difference between Selected and Control populations or between poor and standard diets) by multiplying contrast estimates by their respective pareto factors.

We also used data from all metabolites to explore the quantitative relationship between the plastic response and the evolutionary response. Biologically it would be the most interesting to look at the relationship of the plastic response of the Control lines (i.e., $X_{\text{CTLpoor}} - X_{\text{CTLstd}}$) and the evolved relationship measured on the poor diet ($X_{\text{SELpoor}} - X_{\text{CTLpoor}}$), where X stand for the estimated means of metabolites for conditions indicated by the subscripts. However, both are functions of the metabolite measurements in Control on poor diet. Consequently, the estimated covariance between them is biased by a component equal to $-\sigma^2_{\text{CTLpoor}}$, where σ^2 stands for the error variance of X; this might produce an artifactual negative correlation in the absence of a real relationship. Therefore, we looked instead at the correlation between the main effects of the diet (corresponding to $X_{\text{SELpoor}} + X_{\text{CTLpoor}} - X_{\text{SELstd}} - X_{\text{CTLstd}}$) and the main effect of evolutionary regime (corresponding to $X_{\text{SELpoor}} - X_{\text{CTLpoor}} + X_{\text{SELstd}} - X_{\text{CTLstd}}$). While the two composite variables are functions of the same measurements, their error covariance is $(\sigma^2_{\text{SELpoor}} - \sigma^2_{\text{CTLpoor}} - \sigma^2_{\text{SELstd}} + \sigma^2_{\text{CTLstd}})/4$; it vanishes if the error variances of the different conditions are the same.

Finally, we tested whether Selected and Control populations differ in overall plastic response of metabolome to diet populations by fitting a major axis regression to $X_{\text{CTLpoor}} - X_{\text{CTLstd}}$ and $X_{\text{SELpoor}} - X_{\text{SELstd}}$ (R package smatr (Warton *et al.* 2012)). A systematic difference in the magnitude of response would be detected as a deviation of the major axis slope from 1.

Developmental rate and survival in food enriched with amino acids

In this experiment the larvae of all Selected and Control populations were raised on three diet treatments: the poor diet (same as in experimental evolution), poor diet supplemented with the three branched chain amino acids (BCAA) and poor diet supplemented with three other essential amino acids: tryptophan (W), histidine (H) and lysine (K). The concentration of these amino acids in the poor diet was estimated based on their content in yeast (Piper *et al.* 2017), and we aimed to double their concentrations in the respective treatments. To this end, the BCAA-supplemented diet consisted of the poor diet supplemented with 1.5 mM valine, 1.8 mM leucine and 1.3 mM isoleucine (Sigma-Aldrich, Switzerland). Similarly, the WHK-enriched diet was supplemented with 0.5 mM, 0.8 mM and 1.9 mM of tryptophan, histidine and lysine, respectively.

Each replicate bottle with 40 mL of one of these food media was seeded with 200 eggs and inoculated with feces as described above. Upon emergence, adult males and females were removed and counted once a day until day 26 counted from egg-laying. These data were used to estimate for each replicate egg-to-adult survival probability and the mean developmental rate (mean of the inverse developmental time). From each bottle 10 females were randomly collected on the day of peak emergence (i.e., when the largest number of females emerged). These female samples were frozen and dried at 70°C for 24h before to be weighed as a group to the nearest 1 µg on a precision balance (Mettler Toledo, MT5); sample weight was divided by the number of females weighed (i.e., by 10, or 9 in replicates where a female escaped) to obtain female weight. We also estimated the average growth rate of the larvae in each bottle as ln(female weight/egg weight)/larval developmental time, where egg weight was assumed to be 5 µg (Kolss *et al.* 2009) and the larval developmental time was estimated as the time from egg laying to the day on which the females were collected minus 5 days to account for the time needed for egg development and metamorphosis. This experiment was done in three blocks with one replicate per population, diet treatment and block.

To test for the effect of diet treatment and evolutionary regime on developmental rate, female dry weight and growth rate, we fitted a linear mixed models using lmer package of R with the

evolutionary regime, treatment, sex (for developmental rate only) and their interactions as fixed factors, the replicate population, block and bottle (for developmental rate only) as random factors. The egg counting and sorting was done by two experimenters with three Control and three Selected populations each (the populations-experimenter pairs were kept the same along the three blocks). Because the egg manipulation could affect the eclosion rate, we included experimenter as a random factor in the models mentioned above. The effects were tested with F tests; the degrees of freedom were estimated with the Satterthwaite approximation. For survival, we fitted a generalized mixed model with binomial error distribution and logit link with the number of emerged versus non emerged flies as the response, with the same fixed and random factors as for growth rate. Significance of the fixed effects was tested with likelihood ratio tests using afex package (Singmann *et al.* 2015); pairwise and custom contrasts were estimated and tested with emmeans package in R.

Stable isotope experiment

This experiment aimed to compare accumulation of the heavy stable isotope of nitrogen (^{15}N) as an indicator of the proportion of absorbed amino acids that were subject to deamination followed by excretion of nitrogen as waste. Amino acid deamination process has a slight preference for the common light nitrogen isotope (^{14}N); as a consequence, proteins in tissues become enriched in ^{15}N to a degree that increases with rate of amino-acids deamination (Minagawa and Wada 1984; Peterson and Fry 1987; Gannes *et al.* 1997). Because ^{15}N is naturally rare, in this experiment we used food media containing yeast enriched in ^{15}N, which improved signal to noise ratio.

Food media preparation

In this experiment we used modified versions of standard and poor media that differed from those used in the course of experimental evolution and in the other experiments described above in two ways (Cavigliasso *et al.* 2020). First, they did not contain cornmeal, leaving yeast as the

only source of nitrogen. Second, we used ^{15}N-enriched baker's yeast in addition to dead brewer's yeast used for the usual standard and poor diets. We grew the baker's yeast (starting from a dry form, OD_{600}=100) overnight at 30°C in ^{15}N-enriched SD media (8.5 g yeast nitrogen base without amino acids and ammonium sulfate, 2.5 g normal ammonium sulfate, 2.5 g ^{15}N-labeled ammonium sulfate $(^{15}NH_4)_2SO_4$ and 20 g sucrose per liter of water). This ^{15}N-enriched yeast was collected by removing the supernatant after centrifugation (5 min at 3000g) and stored at -20°C. To prepare "^{15}N-enriched standard diet", we added 2.5 mL of ^{15}N-enriched cultured yeast to a suspension of 12.5 g of dry baker's yeast in 100 mL of water before transferring it in one liter of water solution containing 10 g agar, 90 g of sucrose, 0.5 g $CaCl_2$, 0.5 g $MgSO_4$, 10 mL Nipagin 10%, 6 mL propionic acid and 20 mL ethanol. After solidification, we blended the diet medium and transferred to petri dishes (10 mL per dish of 46 mm diameter). The corresponding "^{15}N-enriched poor diet" was prepared in the analogous way, but one-fourth of the amounts of sugar and yeast of both kinds were used.

Sample collection

To raise individuals for isotope measurements we transferred approximately 150 eggs to petri dishes with ^{15}N-enriched standard or poor diet. For each sample, we collected 20-30 prepupae and 20-30 virgin adult females, pooled from two (standard diet) or three (poor diet) petri dishes; the females were starved for three hours on agar to permit emptying of their guts. The samples were flash-frozen and stored at -80°C. Two samples were collected per population and diet, in two blocks per diet carried out on different dates. Because the level of ^{15}N in diet may differ between block and diet and thus may affect the level in tissue sample, we also collected a "diet sample" for each diet and block.

Isotope measurement

We followed identical protocol to (Cavigliasso *et al.* 2020): The relative ^{15}N content of the samples was determined by elemental analysis/isotope ratio mass spectrometry (EA/IRMS), using a Carlo Erba 1108 (Fisons Instruments, Milan, Italy) elemental analyzer connected to a Delta V Plus isotope ratio mass spectrometer via a ConFlo III split interface (both of Thermo Fisher Scientific, Bremen, Germany), operated under continuous helium (He) flow (Spangenberg *et al.* 2006; Spangenberg *et al.* 2020). The nitrogen stable isotope compositions were reported in the delta (δ) notation as per mil (‰) variations of the molar ratio (R) of the heavy (^{15}N) to light isotope (^{14}N) of nitrogen relative to the international standard molecular nitrogen in air (N_2-Air).

$$\delta^{15}N_{sample/standard} = \frac{R\left(\frac{15\mathrm{N}}{14\mathrm{N}}\right)sample}{R\left(\frac{15\mathrm{N}}{14\mathrm{N}}\right)\mathrm{N2{-}Air}} - 1$$

For calibration and normalization of the measured $\delta^{15}N$ values to N_2-Air scale, a 3- point calibration was used with international reference materials (RMs) and University of Lausanne in-house standards (Spangenberg *et al.* 2020). The nitrogen concentration (in wt.%) were determined from the peak areas of the major isotopes using the calibrations for $\delta^{15}N_{Air\text{-}N2}$. The repeatability was better than 0.2 wt.%.

Statistical analysis

To account for potential variation of $\delta^{15}N$ values between diet types and blocks, $\delta^{15}N$ values in prepupae and adults were normalized by subtracting their $\delta^{15}N$ vales from the corresponding diet and block. These normalized values ($\Delta^{15}N$) were used as the response variable. We fitted a linear mixed model (LMM) with Type 3 F-tests with the selection regime, developmental stage, diet and their interactions as fixed factors, the replicate populations, its interaction with stage and blocks as random factors.

Supplementary Discussion

Caveats on the interpretation of results

We generally interpret genetically-based divergence between Selected and Control populations as a consequence of evolutionary adaptation of the former to the poor larval diet. From this perspective, differences between Selected and Control larvae raised on poor diet would represent a direct response to selection driven by exposure to the poor larval diet, and differences between Selected and Control larvae raised on standard diet would be correlated responses to that selection. This is based on the fact that the base population from which these populations were derived originated from crossing of four populations maintained on the standard diet for 5 years prior to the onset of the evolution experiment . This base population was then maintained for another 7 generations on standard diet following the cross, before the Selected and Control populations were derived (Kolss *et al.* 2009). So from the start, Control populations should have been reasonably well adapted to the standard diet. However, we cannot exclude that they still had some potential to adapt to the standard diet; in particular, new gene combinations potentially improving performance on the standard diet may have been generated by crossing the four populations only seven generations before the start of the experiment. This may have facilitated continuing adaptation of the Control populations to the standard diet after the start of the evolution experiment.

Furthermore, even though most aspects of population maintenance, including the controlled larval density, were the same for the ancestral populations and the Control populations, the Control populations were switched to a 3-week generation cycle (to keep them at synchrony with Selected populations), compared to a 2- to 2.5-week cycle of the ancestral populations. Most Control flies emerge from the standard diet 11-12 days after egg laying. Thus, under the ancestral 2- to 2.5-week maintenance regime, flies were only 2-5 days old when reproducing and only transferred to fresh food with ad libitum yeast one day before collection of eggs for the following generation. Under the Control regime, flies were collected from their larval bottles 14 days after egg laying and spent six days on fresh standard diet before egg collection, i.e., they reproduced

at the age of 9-10 days. These changes in the detailed maintenance protocol may have favored some physiological changes in the Control populations. Selected flies were also transferred to fresh standard diet soon after the peak of eclosion (usually 14 days after egg laying, sometimes later if their development on poor diet was delayed) and thus also spent several days on fresh standard diet before oviposition. However, with the developmental on poor diet being longer, they were several days younger at reproduction than Control flies. This difference at adult age at reproduction may have in principle contributed to differential selection on larval metabolism.

However, in both cases the flies were still at their reproductive prime; thus, any such differential selection due to the difference in the age of reproduction would likely have been weak by comparison to the selection imposed by the strong nutritional stress due to the poor larval diet. This is supported by our finding of larval metabolome differentiation being much more pronounced on the poor than standard diet, as well as a major improvement of the developmental time on the poor diet in Selected compared to Control populations. Thus, while we cannot exclude that evolutionary change in the Control populations contributed to the divergence in larval metabolism between the two sets of populations, we argue that such contribution would be minor compared to adaptation of Selected populations to the novel and physiologically stressful poor diet.

Another caveat related to the history of the ancestral populations concerns the representativeness of the genetic variants underlying dietary adaptation of our Selected populations. The timescale of the evolution experiment and the small population size imply that virtually all of the adaptive evolutionary divergence between Selected and Control populations must have been based on the standing genetic variation that already segregated in the base population (Hermisson and Pennings 2005). This variation had been shaped by the past environmental and demographic conditions, i.e., relatively small populations maintained under constant and benign environmental conditions in the lab. The resulting selection and genetic drift likely reduced the relevant genetic variation to a subset of what would be available in a natural population. Nonetheless, despite this presumably genetically depauperated base population, Selected populations rapidly evolved markedly improved performance on the poor diet (Kolss *et*

al. 2009), and genomic analysis indicated its highly polygenic nature, implicating over 100 genomic regions (Kawecki *et al.* 2021). Thus, genetic variants actually available for dietary adaptation were a rather large sample of such potential variants.

However, this sample may still have been biased by several years (about 100 generations) of selection on the standard diet in the lab. In particular, adaptive plastic responses to variation in diet evolved by the ancestral population in the wild might have eroded. Thus, the absence of relationship between phenotypic plasticity and evolutionary change in our study may be a consequence prior lab adaptation. The fact that despite 240 generations of evolution on different diets the Selected and Control populations still showed very similar plastic responses suggests that such plastic responses do not erode or change rapidly in constant environments. Furthermore, many arguments about the role of phenotypic plasticity as a "driver" of evolutionary change assume that much of plasticity will be adaptive even if the organism is confronted with a completely novel environment (Pigliucci and Murren 2003). Thus, if lab adaptation led to loss of "evolutionary memory" of past exposure to nutrient shortage, the poor diet regime would more genuinely represent a truly novel environment for the Selected populations, and hence our results would remain relevant to the debate about the role of plasticity in evolution.

The above caveats, as well as other limitations are common to laboratory experimental evolution studies, as has been discussed elsewhere (Kawecki *et al.* 2012a). Thus, full understanding of dietary adaptation will necessitate integration of results and insights obtained with diverse approaches available to evolutionary biology (Kawecki *et al.* 2012b).

Supplementary Tables

Supplementary Table S1. Summary of biological hypotheses and specific predictions with their associated results and corresponding figures.

**Biological hypothesis**	**Specific prediction(s)**	**Conclusion**	**Figure**
Adaptation to larval undernutrition is associated with major changes in metabolism	Patterns of metabolite abundance differ markedly between Selected and Control larvae, in particular when raised on poor diet	Yes	1, 2
Phenotypic plasticity predicts evolutionary change	Overlap between metabolites significant for the diet treatment and those significant for regime greater than expected by chance; changes due to regime (SEL – CTL) positively correlated with changes due to diet (poor – standard)	No	2,3B
Evolution on poor diet largely restores metabolic state to ancestral state on standard diet	Evolution in Selected populations reverts most plastic changes induced by poor diet in Control larvae; metabolic state of Selected larvae on poor diet resembles that of Controls on standard diet	No	3A
Adaptation to poor diet increases robustness to diet change	The plastic response of Selected larvae to diet is less strong than that of Controls	Yes (slightly)	3C
Low level of BCAA is advantageous on poor diet	Supplementation of BCAA will reduce larval survival or impair development	No	4E,F
BCAA are limiting for growth on poor diet	Supplementation of BCAA will results in faster growth and development of both Control and Selected larvae, supplementation of WHK will not	Yes	4F-H
Undernutrition-adapted larvae use a greater fraction of amino acids for energy generation	Selected larvae show higher concentrations of urate and its precursors than Control larvae	Yes	2, 5A,B
	Selected larvae accumulate higher levels of ^{15}N than Controls	Yes	5C

Supplementary Table S2. Summary of results of the ANOVAs on scores from the first three principal components of metabolite abundance.

Factor	PC1		PC2		PC3	
	Statistics	*P*	Statistics	*P*	Statistics	*P*
Regime	$F_1 = 0.1$	0.71	$F_1 = 14.3$	**0.001**	$F_1 = 13.7$	**0.001**
Diet	$F_1 = 1274.7$	**< 0.001**	$F_1 = 0.2$	0.68	$F_1 = 0.1$	0.82
Regime × Diet	$F_1 = 2.1$	0.17	$F_1 = 1.7$	0.21	$F_1 = 1.2$	0.29

Supplementary Table S3. Summary of univariate analysis with q-values and log2 fold change. A separate file available at
http://academic.oup.com/evlett/article-lookup/doi/10.1093/evlett/qrad018#supplementary-data .

Supplementary Table S4. Significance tests from linear mixed models (LMM) for the four amino acids that show significant diet × regime interaction: BCAA (leucine, isoleucine and valine) and proline, reported in Figure 4A-D. SEL: Selected populations; CTL: Control populations; Std: standard diet. *q* are the corrected *P* (10% FDR).

Factors	Leucine		Isoleucine		Valine		Proline	
	Statistics	*q*	Statistics	*q*	Statistics	*q*	Statistics	*q*
Regime	$F_{1,13} = 6.9$	**0.061**	$F_{1,12} = 8.6$	**0.044**	$F_{1,12} = 5.8$	**0.073**	$F_{1,12} = 1.1$	0.31
Diet	$F_{1,54} = 0.2$	0.70	$F_{1,5} = 2.6$	0.17	$F_{1,6} = 7.2$	**0.038**	$F_{1,4} = 10.2$	**0.031**
Regime × Diet	$F_{1,54} = 14.3$	**0.018**	$F_{1,48} = 16.7$	**0.011**	$F_{1,49} = 18.4$	**0.011**	$F_{1,12} = 13.8$	**0.071**
Pairwise Contrasts								
SEL – CTL in poor diet	$t_{25} = 4.1$	**0.004**	$t_{24} = 4.4$	**0.003**	$t_{24} = 4.4$	**0.003**	$t_{20} = 2.3$	**0.073**
SEL – CTL in std diet	$t_{24} = 0.3$	0.77	$t_{23} = 0.0$	0.96	$t_{23} = 0.9$	0.56	$t_{20} = 0.5$	0.62
Poor – Std in SEL	$t_6 = 2.8$	**0.051**	$t_{12} = 3.5$	**0.014**	$t_{13} = 4.1$	**0.006**	$t_{14} = 0.1$	0.95
Poor – Std in CTL	$t_6 = 2.3$	**0.057**	$t_{11} = 1.4$	0.19	$t_{13} = 0.3$	0.79	$t_{14} = 4.2$	**0.002**

Supplementary Table S5: Amino acid supplementation experiment: summary of significance tests from generalized linear mixed model for egg-to-adult survival rate reported in Figure 4E. Treatment refers to the three types of diet treatment (poor diet, BCAA supplementation, WHK supplementation).

Factors	Statistics	*P*
Regime	$\chi^2 = 1.5$	0.21
Treatment	$\chi^2 = 3.4$	0.18
Regime × Treatment	$\chi^2 = 0.1$	0.97

Supplementary Table S6. Amino acid supplementation experiment: summary of significance tests from LMM for developmental rate reported in Figure 4F. Treatment refers to the three types of diet treatment (poor diet, BCAA supplementation, WHK supplementation).

Factors	Statistics	*P*
Regime	$F_{1,9} = 245.5$	< 0.001
Treatment	$F_{2,91} = 36.8$	< 0.001
Sex	$F_{1,10} = 3.9$	0.075
Regime × Treatment	$F_{2,91} = 4.2$	0.018
Regime × Sex	$F_{1,10} = 1.0$	0.34
Treatment × Sex	$F_{2,22} = 1.2$	0.32
Regime × Treatment × Sex	$F_{2,22} = 3.6$	0.046
Pairwise Contrasts		
For Control		
BCAA – Poor	$t_{21} = 3.5$	0.006
WHK – Poor	$t_{20} = -0.4$	0.90
BCAA – WHK	$t_{21} = 4.0$	0.002
For Selected		
BCAA – Poor	$t_{20} = 4.6$	< 0.001
WHK – Poor	$t_{20} = 3.4$	0.008
BCAA – WHK	$t_{20} = 8.0$	< 0.001
Custom Interaction Contrasts		
$(BCAA – Poor)_{in\ SEL}$ – $(BCAA – Poor)_{in\ CTL}$	$t_{20} = 0.7$	0.48
$(WHK – Poor)_{in\ SEL}$ – $(WHK – Poor)_{in\ CTL}$	$t_{20} = -2.1$	0.051

Supplementary Table S7: Amino acid supplementation experiment: summary of significance tests from LMM for female weight reported in Figure 4G. Treatment refers to the three types of diet treatment (poor diet, BCAA supplementation, WHK supplementation).

Factors	Statistics	*P*
Regime	$F_{1,10} = 88.3$	< 0.001
Treatment	$F_{2,20} = 2.0$	0.16
Regime × Treatment	$F_{2,20} = 1.8$	0.19

Supplementary Table S8. Amino acid supplementation experiment: summary of significance tests from LMM for female growth rate reported in Figure 4H. Treatment refers to the three types of diet treatment (poor diet, BCAA supplementation, WHK supplementation).

Factors	Statistics	*P*
Regime	$F_{1,9} = 21.5$	0.001
Treatment	$F_{2,20} = 9.0$	0.002
Regime × Treatment	$F_{2,20} = 1.4$	0.26
Pairwise comparisons		
BCAA – Poor	$t_{20} = 2.8$	0.029
WHK – Poor	$t_{20} = -1.4$	0.36
BCAA – WHK	$t_{20} = 4.2$	0.001

Supplementary Table S9. Summary of type 3 F-tests with Satterthwaite's method for fixed effect in LMM on $\Delta^{15}N$ (‰ N_2-Air) reported in Figure 5C.

Factors	F value	*P*
Regime	$F_{1,11} = 18.9$	0.001
Stage	$F_{1,71} = 18.4$	<0.001
Diet	$F_{1,12} = 88.4$	<0.001
Regime × Stage	$F_{1,71} = 1.5$	0.22
Regime × Diet	$F_{1,12} = 2.8$	0.12
Diet × Stage	$F_{1,71} = 0.9$	0.34
Regime × Stage × Diet	$F_{1,71} = 0.2$	0.63

Supplementary Figures

Supplementary Figure S1. 3D animation of sample clustering using the first 3 principal components of multiparametric metabolic profiles composed of 174 metabolite abundance data; each point corresponds to a population estimate averaged over replicate samples. A separate file available at http://academic.oup.com/evlett/article-lookup/doi/10.1093/evlett/qrad018#supplementary-data .

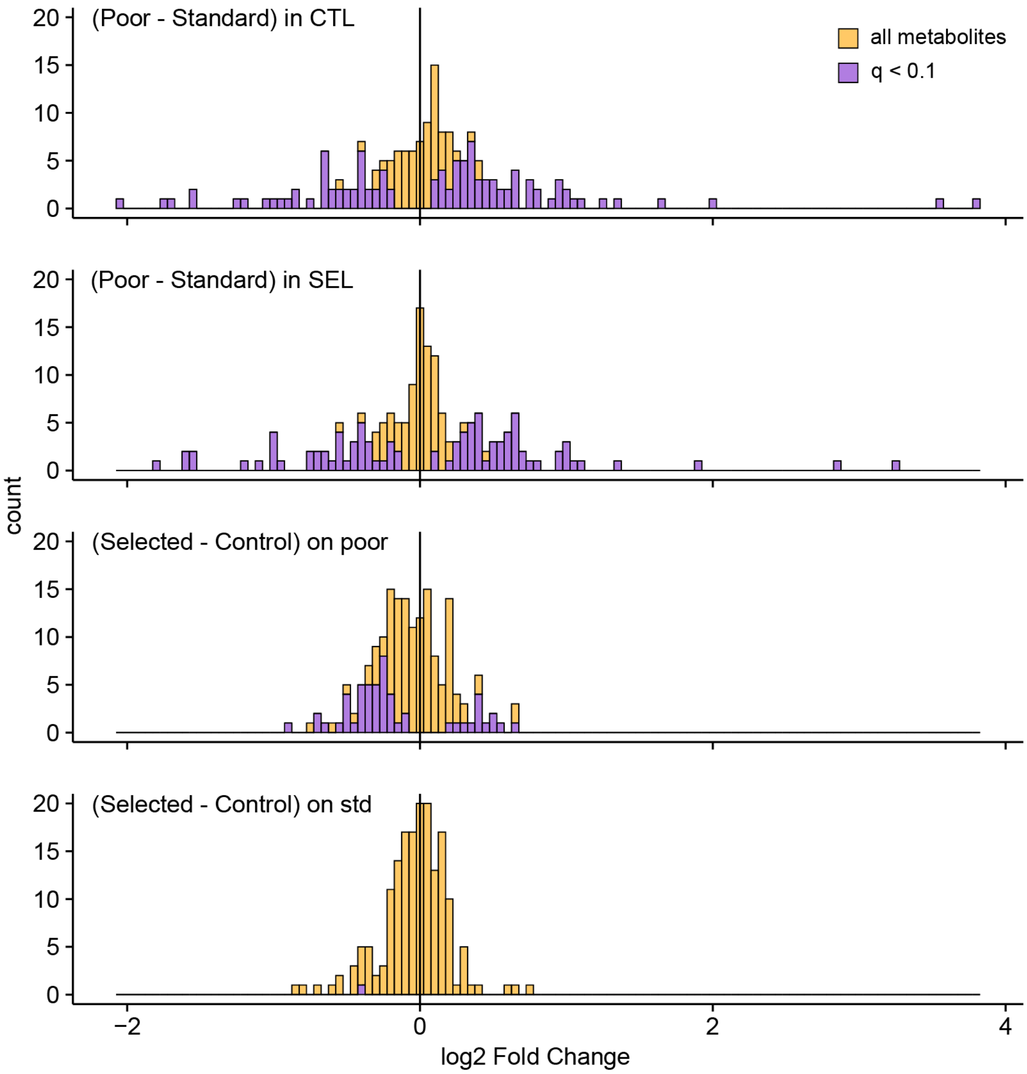

Supplementary Figure S2: Distribution of differences in metabolite abundance for different pairwise contrasts. CTL: Control; SEL: Selected; poor: poor diet; std: standard diet. Purple bars: metabolites significant ($q < 0.1$) for the particular contrast.

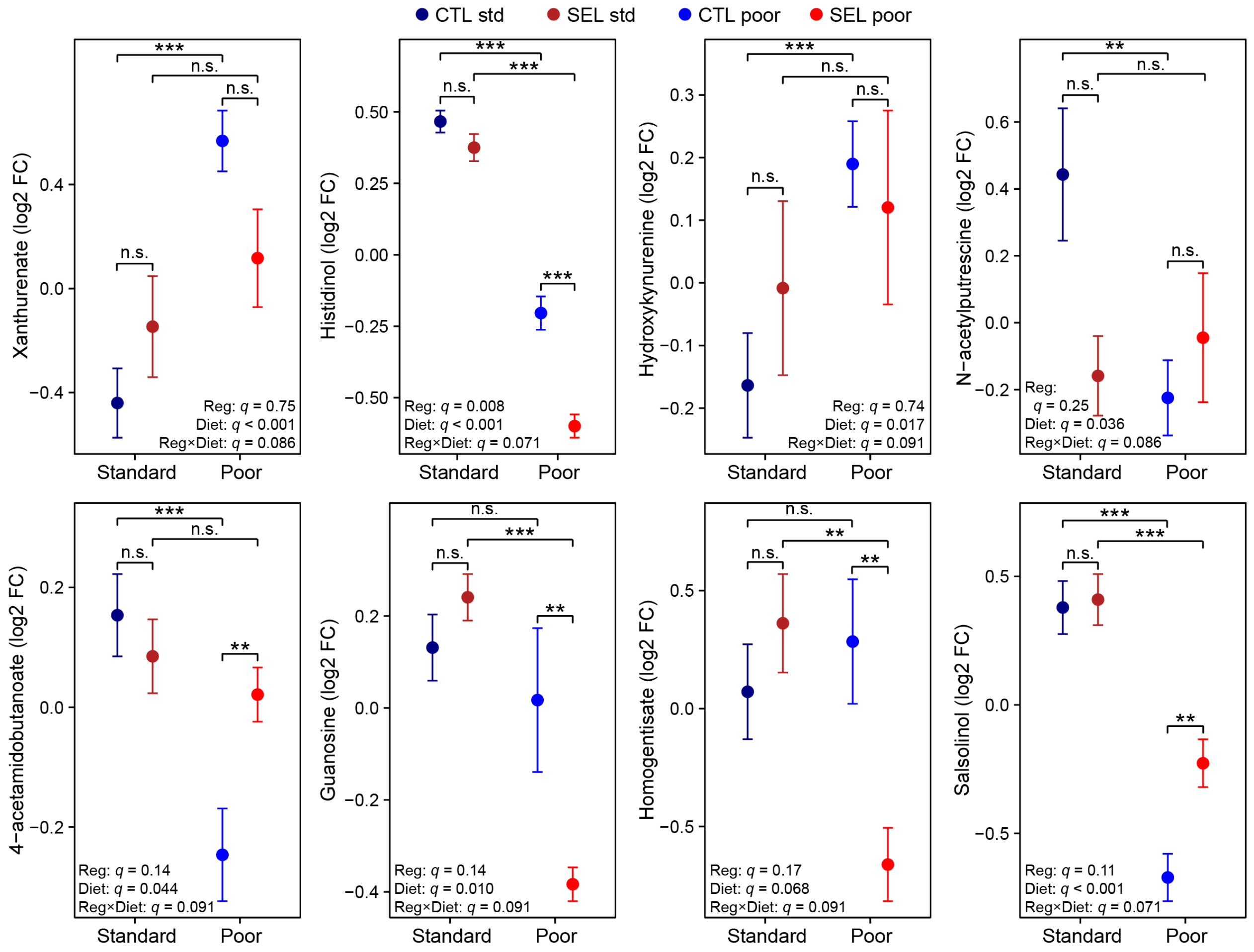

Supplementary Figure S3. Relative abundance of metabolites with significant regime × diet interaction (*q* < 0.1) in Selected (SEL) and Control (CTL) larvae raised on poor and standard (std) diet. Level of significance: n.s not significant *q* > 0.1; * *q* < 0.1; ** *q* < 0.05; *** *q* < 0.01. Symbols indicate means ± SE. N = 3 batches (pool of 15-20 larvae) per population and diet. Reg: evolutionary regime.

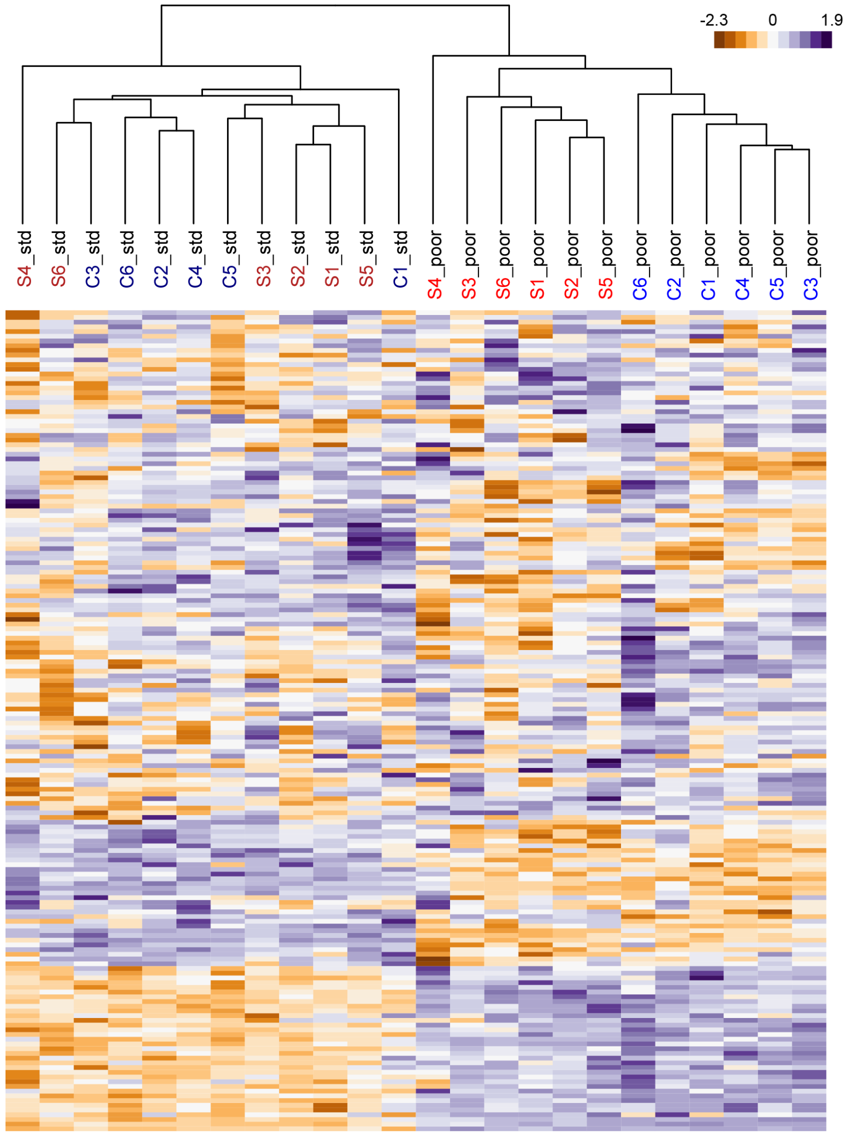

Supplementary Figure S4: Clustering of populations based on their metabolic profiles. Each cell of the heat map is colored according to the metabolite abundance (estimate averaged over replicate samples, as for the PCA in Figure 1A). S1-S6: Selected populations, C1-C6: Control populations; "_poor": larvae raised on poor diet, "_std": larvae raised on standard diet.

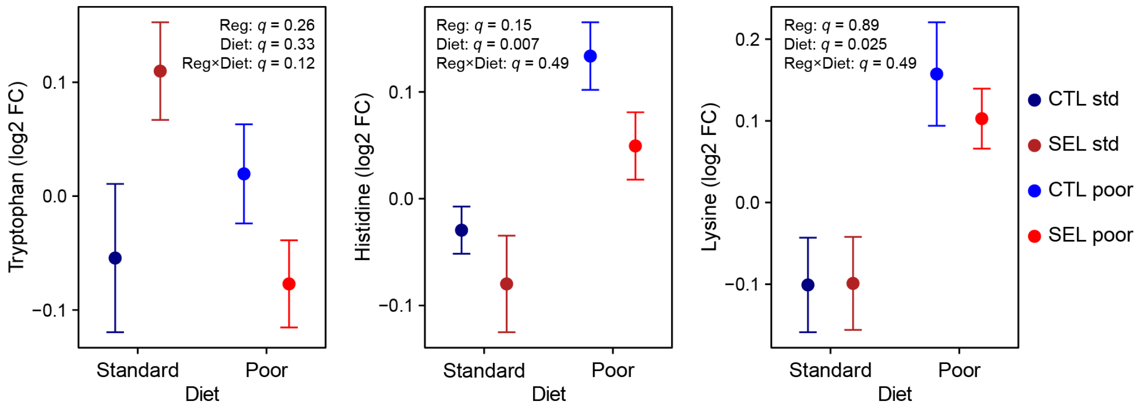

Supplementary Figure S5. Relative amount of tryptophan, histidine and lysine in Selected (SEL) and Control (CTL) larvae raised on poor and standard (std) diet. Symbols indicate means ± SE. N = 3 batches (pool of 15-20 larvae) per population and diet. Reg: evolutionary regime.

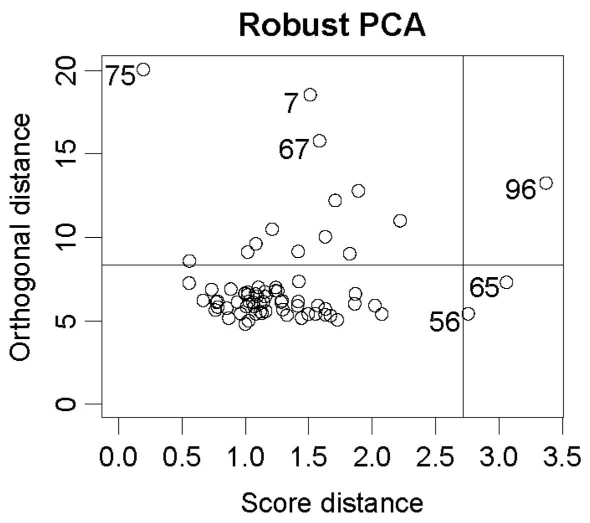

Supplementary Figure S6. Robust PCA fitted with two components. Based on visual inspection, six outlier samples (labeled with sample number) were removed for the analysis. The two lines show the conventionally assumed cutoffs (for details see (Hubert *et al.* 2005)).

References

Broadhurst, D., Goodacre, R., Reinke, S.N., Kuligowski, J., Wilson, I.D., Lewis, M.R. *et al.* 2018. Guidelines and considerations for the use of system suitability and quality control samples in mass spectrometry assays applied in untargeted clinical metabolomic studies. *Metabolomics*, 14, 72.

Cavigliasso, F., Dupuis, C., Savary, L., Spangenberg, J.E. and Kawecki, T.J. 2020. Experimental evolution of post-ingestive nutritional compensation in response to a nutrient-poor diet. *Proc. Royal Soc. B*, 287, 20202684.

Cox, J.E., Thummel, C.S. and Tennessen, J.M. 2017. Metabolomic studies in *Drosophila*. *Genetics*, 206, 1169-1185.

Dunn, W.B., Broadhurst, D., Begley, P., Zelena, E., Francis-McIntyre, S., Anderson, N. *et al.* 2011. Procedures for large-scale metabolic profiling of serum and plasma using gas chromatography and liquid chromatography coupled to mass spectrometry. *Nat. Protoc.*, 6, 1060-1083.

Gannes, L.Z., O'Brien, D.M. and Del Rio, C.M. 1997. Stable isotopes in animal ecology: assumptions, caveats, and a call for more laboratory experiments. *Ecology*, 78, 1271-1276.

Hermisson, J. and Pennings, P.S. 2005. Soft sweeps: molecular population genetics of adaptation from standing genetic variation. *Genetics*, 169, 2335-2352.

Hubert, M., Rousseeuw, P.J. and Vanden Branden, K. 2005. ROBPCA: A new approach to robust principal component analysis. *Technometrics*, 47, 64-79.

Kannangara, J.R., Mirth, C.K. and Warr, C.G. 2021. Regulation of ecdysone production in *Drosophila* by neuropeptides and peptide hormones. *Open Biol.*, 11, 200373.

Kawecki, T.J., Erkosar, B., Dupuis, C., Hollis, B., Stillwell, R.C. and Kapun, M. 2021. The genomic architecture of adaptation to larval malnutrition points to a trade-off with adult starvation resistance in *Drosophila*. *Mol. Biol. Evol.*, 38, 2732-2749.

Kawecki, T.J., Lenski, R.E., Ebert, D., Hollis, B., Olivieri, I. and Whitlock, M.C. 2012a. Experimental evolution. *Trends Ecol. Evol.*, 27, 547-560.

Kawecki, T.J., Lenski, R.E., Ebert, D., Hollis, B., Olivieri, I. and Whitlock, M.C. 2012b. The value of complementary approaches in evolutionary research: reply to Magalhães and Matos. *Trends Ecol. Evol.*, 27, 650-651.

Kolss, M., Vijendravarma, R.K., Schwaller, G. and Kawecki, T.J. 2009. Life-history consequences of adaptation to larval nutritional stress in *Drosophila*. *Evolution*, 63, 2389-2401.

Kuznetsova, A., Brockhoff, P.B. and Christensen, R.H.B. 2017. lmerTest Package: tests in linear mixed effects models. *J. Stat. Softw.*, 82.

Lavrynenko, O., Rodenfels, J., Carvalho, M., Dye, N.A., Lafont, R., Eaton, S. *et al.* 2015. The ecdysteroidome of *Drosophila*: influence of diet and development. *Development*, 142, 3758-3768.

Li, H. and Tennessen, J.M. 2018. Preparation of *Drosophila* larval samples for gas chromatography-mass spectrometry (GC-MS)-based metabolomics. *J. Vis. Exp.*

Medina, J., van der Velpen, V., Teav, T., Guitton, Y., Gallart-Ayala, H. and Ivanisevic, J. 2020. Single-step extraction coupled with targeted HILIC-MS/MS approach for comprehensive analysis of human plasma lipidome and polar metabolome. *Metabolites*, 10, 495.

Minagawa, M. and Wada, E. 1984. Stepwise enrichment of 15N along food chains: further evidence and the relation between δ15N and animal age. *Geochim. Cosmochim. Acta*, 48, 1135-1140.

Peterson, B.J. and Fry, B. 1987. Stable isotopes in ecosystem studies. *Annu. Rev. Ecol. Syst.*, 18, 293-320.

Pigliucci, M. and Murren, C.J. 2003. Genetic assimilation and a possible evolutionary paradox: can macroevolution sometimes be so fast as to pass us by? *Evolution*, 57, 1455-1464.

Piper, M.D.W., Soultoukis, G.A., Blanc, E., Mesaros, A., Herbert, S.L., Juricic, P. *et al.* 2017. Matching dietary amino acid balance to the in silico-translated exome optimizes growth and reproduction without cost to lifespan. *Cell Metab.*, 25, 1206.

Rohart, F., Gautier, B., Singh, A. and Lê Cao, K.-A. 2017. mixOmics: An R package for ‘omics feature selection and multiple data integration. *PLOS Comput. Biol.*, 13, e1005752.

Singmann, H., Bolker, B., Westfall, J., Aust, F. and Ben-Shachar, M.S. 2015. afex: analysis of factorial experiments. *R package version 0.13–145*.

Spangenberg, J.E., Jacomet, S. and Schibler, J. 2006. Chemical analyses of organic residues in archaeological pottery from Arbon Bleiche 3, Switzerland–evidence for dairying in the late Neolithic. *J. Archaeol. Sci.*, 33, 1-13.

Spangenberg, J.E., Schweizer, M. and Zufferey, V. 2020. Shifts in carbon and nitrogen stable isotope composition and epicuticular lipids in leaves reflect early water-stress in vineyards. *Sci. Total Environ.*, 739, 140343.

Storey, J.D. and Tibshirani, R. 2003. Statistical significance for genomewide studies. *Proc. Natl. Acad. Sci. U.S.A.*, 100, 9440-9445.

Tsugawa, H., Kanazawa, M., Ogiwara, A. and Arita, M. 2014. MRMPROBS suite for metabolomics using large-scale MRM assays. *Bioinformatics*, 30, 2379-2380.

van der Velpen, V., Teav, T., Gallart-Ayala, H., Mehl, F., Konz, I., Clark, C. *et al.* 2019. Systemic and central nervous system metabolic alterations in Alzheimer's disease. *Alzheimer's Res. Ther.*, 11, 93.

Warton, D.I., Duursma, R.A., Falster, D.S. and Taskinen, S. 2012. smatr 3 - an R package for estimation and inference about allometric lines. *Methods Ecol. Evol.*, 3, 257-259.
