# Supplementary material for: Experimental evolution of metabolism under nutrient restriction: enhanced amino acid catabolism and a key role of branched-chain amino acids
Source: Evol Lett. 2023 May 18;7(4):273–84. doi: 10.1093/evlett/qrad018 (PMC10355184; doi:10.1093/evlett/qrad018)
Supplement: qrad018_suppl_Supplementary_Data [file qrad018_suppl_supplementary_data.zip › script metabolomics BCAA_WHK 15N_enrichment.pdf]

```
library(ggplot2)
library(ggpubr)
library(mixOmics)
library(lmerTest)
library(emmeans)
library(rrcov)
library(redres)
library(rlist)
library(sjstats)
library(afex)
library(car)

################################################################################
############################                ######################################
############################ METABOLOMICS ######################################
############################                ######################################
################################################################################

#########################
#### Outlier removal ####
#######################

read.table("metabo_allsamples_pareto",h=T)->my_data
head(my_data)
my_data$sample
paste(my_data$diet,my_data$regime,sep="_")->my_data$diet_reg
head(my_data)
dim(my_data)# 71 183
metabo<-my_data[,-c(1:7,182,183)]
class_grp<-my_data$diet_reg

# PCA robust #
pca2 <- PcaHubert(metabo,k = 2,mcd=T)
pca2
plot(pca2)
# Visual observation: outliers are observation #7, 38, 47, 48, 56 and 63 (correspond to sample name #7,
56, 65, 67, 75 and 96)
my_data[c(7, 38, 47, 48,56,63),c(1)]

# studentized residuals #
head(my_data)
my_data<-my_data[-c(7, 38, 47, 48,56,63),]
my_data$block<-factor(my_data$batch)
my_data$population<-factor(my_data$population)
```

```
resultsList_StudentResid2 <- list()
for (i in 8:181) {
  lmfit_i <- lmer(my_data[,i]~regime*diet+ (1| population) +(1|diet:population)+(1|batch), data
=my_data,REML=F,control=lmerControl(optimizer="bobyqa",optCtrl=list(maxfun=1e9)))
  metabo_name_i<-rep(colnames(my_data)[i],65)
  metabo_num_i<-rep(i,65)
  rc_resids_i <- compute_redres(lmfit_i ,type = "std_cond")
  metato_i<-cbind(metabo_num_i,my_data$sample,metabo_name_i,rc_resids_i,abs(rc_resids_i))
  resultsList_StudentResid2[[i]] <- metato_i
}

lapply(resultsList_StudentResid2, function(x) write.table( data.frame(x), "studentResid_real_pareto.txt",
append= T, sep="," ))


########################################
#### New analysis on clean dataset ####
########################################

#### New GLMM and ANOVA after metabolit-specific removal ####
read.table("metabol_outliersRM.txt",h=T)->my_data_clean
head(my_data_clean)
dim(my_data_clean) #65, 182
my_data_clean$block<-factor(my_data_clean$batch)
my_data_clean$population<-factor(my_data_clean$population)
my_data_clean$regime<-factor(my_data_clean$regime)
my_data_clean$diet<-factor(my_data_clean$diet)

# to get estimate to calculate fold change
resultsList_FC <- list()
for (i in 8:184) {
  lmfit_i <- lmer(my_data_clean[,i]~regime*diet+ (1| population) +(1|diet:population)+(1|batch), data
=my_data_clean,REML=F,control=lmerControl(optimizer="bobyqa",optCtrl=list(maxfun=1e9)))
  ls_mean_i<-ls_means(lmfit_i,which = "regime:diet", pairwise = F)
  resultsList_FC[[i]] <- cbind(colnames(my_data_clean)[i],t(ls_mean_i$Estimat))
}

lapply(resultsList_FC, function(x) write.table( data.frame(x), "estimate_FC.txt", append= T, sep=";" ))


# to get main effect output
resultsList <- list()
for (i in 8:184) {
```

```
  lmfit_i <- lmer(my_data_clean[,i]~regime*diet+ (1| population) +(1|diet:population)+(1|batch), data
=my_data_clean,REML=F,control=lmerControl(optimizer="bobyqa",optCtrl=list(maxfun=1e9)))
  resultsList[[i]] <- anova(lmfit_i)
}

lapply(resultsList, function(x) write.table( data.frame(x), "anova_output.txt", append= T, sep="," ))


#### contrasts ####

resultsList_contrast <- list()
for (i in 8:184) {
  lmfit_i <- lmer(my_data_clean[,i]~regime*diet+ (1| population) +(1|diet:population)+(1|batch), data
=my_data_clean,REML=F,control=lmerControl(optimizer="bobyqa",optCtrl=list(maxfun=1e9)))
  lmfit_i <- emmeans(lmfit_i,"regime",by=c("diet"))
  resultsList_contrast[[i]]<-pairs(lmfit_i)
  }

lapply(resultsList_contrast, function(x) write.table( data.frame(x), "contrasts_regime2.txt", append= T,
sep="," ))

resultsList_contrast_diet <- list()
for (i in 8:181) {
  lmfit_i <- lmer(my_data_clean[,i]~regime*diet+ (1| population) +(1|diet:population)+(1|batch), data
=my_data_clean,REML=F,control=lmerControl(optimizer="bobyqa",optCtrl=list(maxfun=1e9)))
  lmfit_i <- emmeans(lmfit_i,"diet",by=c("regime"))
  resultsList_contrast_diet[[i]]<-pairs(lmfit_i)
  }

lapply(resultsList_contrast_diet, function(x) write.table( data.frame(x), "contrasts_diet2.txt", append= T,
sep="," ))

# specific contrasts : SEl_poor - CTL_std #
resultsList_contrast_spec <- list()
for (i in 8:181) {
  lmfit_i <- lmer(my_data_clean[,i]~regime*diet+ (1| population) +(1|diet:population)+(1|batch), data
=my_data_clean,REML=F,control=lmerControl(optimizer="bobyqa",optCtrl=list(maxfun=1e9)))
  lmfit_i <- emmeans(lmfit_i,"regime",by=c("diet"))
  SEL_poor=c(1,0,0,0)
  CTL_std=c(0,0,0,1)
  resultsList_contrast_spec[[i]]<-contrast(lmfit_i, method = list(SEL_poor - CTL_std) ,by = NULL)
  }
```

```
lapply(resultsList_contrast_spec, function(x) write.table( data.frame(x), "contrasts_homeostasis.txt",
append= T, sep="," ))


#################################
##### Multivariate analysis #####
#################################

head(my_data_clean)
metabo_clean<-my_data_clean[,-c(1:7,182,183)]
class_grp<-my_data_clean$diet_reg

## PCA on pop (average) ##
### Model to extract emmeans estimate to be used as imput for PCA ###
head(my_data_clean)
estimate_<-NULL
for (i in 8:181) {
  lmfit_i <- lmer(my_data_clean[,i]~population*diet+(1|batch), data
=my_data_clean,REML=F,control=lmerControl(optimizer="bobyqa",optCtrl=list(maxfun=1e9)))
  lmfit_i <- emmeans(lmfit_i,"population",by=c("diet"))
  lmfit_i_bis<-summary(lmfit_i)
  estimate_[[i]]<-lmfit_i_bis[,c(3)]
}
list.cbind(estimate_)->estimates_column
head(estimates_column)
write.table(estimates_column,"estimate_pop_diet_column.txt", sep=";")


read.table("average_estimated_for_PCA.txt",h=T)->mean_estimates
head(mean_estimates)
dim(mean_estimates) #24, 177
mean_estimates$population<-factor(mean_estimates$population)
paste(mean_estimates$diet,mean_estimates$regime,sep="_")->mean_estimates$diet_reg
paste(mean_estimates$diet,mean_estimates$population,sep="_")->mean_estimates$diet_pop
str(mean_estimates)

metabo_mean<-mean_estimates[,-c(1:3,178,179)]
class_grp<-mean_estimates$diet_reg

pca.res2 = pca(metabo_mean, ncomp = 10,center = T,scale = F)
pca.res2$sdev

library(rgl)
library(dplyr)
```

```
mean_estimates$diet_reg2<-paste(mean_estimates$diet,mean_estimates$regime)

mean_estimates$diet_reg2<-recode_factor(mean_estimates$diet_reg2,
                                "poor Control"="Ctrl on poor diet",
                                "poor 1Selected"="Sel on poor diet",
                                "std Control"="Ctrl on std diet",
                                "std 1Selected"="Sel on std diet")

pca_mean_pop_PC123<-plotIndiv(pca.res2, group = mean_estimates$diet_reg2,
                        col.per.group = c("blue","red","navy","firebrick"),
                        legend = TRUE, title = 'PCA mean pop',
                        ellipse = F,ellipse.level=0.95,
                        ind.names = mean_estimates$diet,
                        pch = "sphere",
                        comp = c(1,2,3),
                        style = "3d",legend.position = "top",
                        X.label = "PC1: 45% expl. var (Diet***)",
                        Y.label = "PC2: 10% expl. var (Reg**)",
                        Z.label = "PC3: 8% expl. var (Reg**)")

pca.res2$variates$X
pca.res2$call
dim(mean_estimates)
head(mean_estimates)
cbind(mean_estimates[,c(1:3)],pca.res2$variates$X)->my_data_scores
head(my_data_scores)

manov2<-manova(cbind(PC1,PC2,PC3)  ~  regime * diet,data=my_data_scores )
summary.aov(manov2) # anova on each PC

summary(manov2,test = "Wilks") # manova on 3 PC

emmeans(manov2,"regime",by=c("diet"))->manov_2
pairs(manov_2) # contrast regime on each diet


##################################
#### Fold change comparisons ####
#################################

#### Correlation between Log2FC ####
library(smatr)
# data: log2 fold change: estimates x pareto scale factor
```

```
# Figure 3B
sma(log2FC_regime~log2FC_diet, data=plasticity_selection, method = "MA",slope.test = 0)

# Figure 3C
sma(log2FC_Diet_in_Sel~log2FC_diet_in_Ctrl, data=plasticity_selection, method = "MA",slope.test = 1)


# comparisons of estimates (median)
head(plasticity_selection)
abs(plasticity_selection$log2FC_regime)->plasticity_selection$log2FC_regime_abs
abs(plasticity_selection$log2FC_diet)->plasticity_selection$log2FC_diet_abs
abs(plasticity_selection$log2FC_Reg_in_poor)->plasticity_selection$log2FC_Reg_in_poor_abs
abs(plasticity_selection$log2FC_Reg_in_std)->plasticity_selection$log2FC_Reg_in_std_abs

median(plasticity_selection$log2FC_regime_abs)
median(plasticity_selection$log2FC_diet_abs)
median(plasticity_selection$log2FC_Reg_in_poor_abs)
median(plasticity_selection$log2FC_Reg_in_std_abs)

wilcox.test(plasticity_selection$log2FC_regime_abs,plasticity_selection$log2FC_diet_abs)
wilcox.test(plasticity_selection$log2FC_regime,plasticity_selection$log2FC_diet)
wilcox.test(plasticity_selection$log2FC_Reg_in_poor_abs,plasticity_selection$log2FC_diet_abs)
wilcox.test(plasticity_selection$log2FC_Reg_in_poor_abs,plasticity_selection$log2FC_Reg_in_std_abs)


#####################################################################################
##############################                ########################################
############################## BCAA and WHK #######################################
##############################                ########################################
#####################################################################################

overdisp_fun <- function(model) {
  ## number of variance parameters in
  ##   an n-by-n variance-covariance matrix
  vpars <- function(m) {
    nrow(m)*(nrow(m)+1)/2
  }
  model.df <- sum(sapply(VarCorr(model),vpars))+length(fixef(model))
  rdf <- nrow(model.frame(model))-model.df
  rp <- residuals(model,type="pearson")
  Pearson.chisq <- sum(rp^2)
  prat <- Pearson.chisq/rdf
  pval <- pchisq(Pearson.chisq, df=rdf, lower.tail=FALSE)
  c(chisq=Pearson.chisq,ratio=prat,rdf=rdf,p=pval)}
```

```
err.std <- function(vecteur) { sd(vecteur)/sqrt(length(vecteur)) }
data_summary <- function(data, varname, groupnames){
  require(plyr)
  summary_func <- function(x, col){
    c(mean = mean(x[[col]], na.rm=TRUE),
      sd = sd(x[[col]], na.rm=TRUE),
      se = err.std(x[[col]]))
  }
  data_sum<-ddply(data, groupnames, .fun=summary_func,
                  varname)
  data_sum <- rename(data_sum, c("mean" = varname))
  return(data_sum)
}

data_sum_1 <- function(data, varname, groupnames){
  require(plyr)
  summary_func <- function(x, col){
    c(sum = sum(x[[col]], na.rm=TRUE))
  }
  data_sum<-ddply(data, groupnames, .fun=summary_func,
                  varname)
  data_sum <- rename(data_sum, c("sum" = varname))
  return(data_sum)}


#####################
### Survival rate ###
#####################
read.table("Raw_data_BCAA and WHK supplementation.txt",h=T)->BCAA_data
head(BCAA_data)

BCAA_data$bottle<-factor(BCAA_data$bottle)
BCAA_data$treatment<-factor(BCAA_data$treatment, levels = c("poor","BCAA","WHK"))
BCAA_data$Pop<-factor(BCAA_data$Pop)
BCAA_data$block<-factor(BCAA_data$block)

droplevels(subset(BCAA_data,!bottle=="83"))->BCAA_data2 # bottle with no emergence due to feces
inoculation issue

BCAA_total<-data_sum_1(BCAA_data2,varname = "Total_nb",
                      groupnames =
c("bottle","Pop","block","treatment","experimenter","regime","Pop_treatment"))

200-BCAA_total$Total_nb->BCAA_total$dead
```

```
##### Analysis ######
head(BCAA_total)

glmer(cbind(Total_nb,dead)~regime*treatment+ (1|experimenter)+(1| Pop)
+(1|treatment:Pop)+(1|block), family = "binomial",data =BCAA_total)->surv_1
overdisp_fun(surv_1) # overdispersion
BCAA_total$obs <- 1:nrow(BCAA_total)

glmer(cbind(Total_nb,dead)~regime*treatment+ (1|experimenter)+ (1| Pop)
+(1|treatment:Pop)+(1|block)+(1|obs),
    family = "binomial",data =BCAA_total,control=glmerControl(optimizer="bobyqa",
optCtrl=list(maxfun=100000)))->surv_1_bis
overdisp_fun(surv_1_bis) # ok
mixed(cbind(Total_nb,dead)~regime*treatment+ (1|experimenter)+ (1| Pop)
+(1|treatment:Pop)+(1|block)+(1|obs),
    family = "binomial", method="LRT",data =BCAA_total,control=glmerControl(optimizer="bobyqa",
optCtrl=list(maxfun=100000)))->surv_2_bis
anova(surv_2_bis)


###################################
### Developmental time (1/day) ###
###################################
head(BCAA_data2)

emergence1<-c(10:26)
emergence1

apply(BCAA_data2[,9:25],1,function(x) weighted.mean(1/emergence1,x))->BCAA_data2$mean_dev_rate

##### Analysis ######
head(BCAA_data2)

lmer(mean_dev_rate ~regime*treatment*sex+ (1|experimenter)+(1| Pop)
+(1|treatment:Pop)+(1|sex:Pop)+(1|sex:treatment:Pop) +  (1|block)+  (1|bottle), data =BCAA_data2)-
>lm_dev_rate_mean_bis
qqnorm(resid(lm_dev_rate_mean_bis))
qqline(resid(lm_dev_rate_mean_bis))
shapiro.test(resid(lm_dev_rate_mean_bis))
anova(lm_dev_rate_mean_bis)

emmeans(lm_dev_rate_mean_bis,"treatment",by=c("regime"))->lm_dev_rate_mean2
pairs(lm_dev_rate_mean2)
```

```
emmeans(lm_dev_rate_mean_bis,"treatment",by=c("regime"))->lm_dev_rate_mean2
lm_dev_rate_mean2

W_P_Sel = c(0,0,0,-1,0,1)
W_P_Ctrl = c(-1,0,1,0,0,0)
BCAA_P_Sel = c(0,0,0,-1,1,0)
BCAA_P_Ctrl = c(-1,1,0,0,0,0)

contrast(lm_dev_rate_mean2, method = list("Test WHK"=W_P_Sel-W_P_Ctrl,
                                  "Test BCAA"=BCAA_P_Sel-BCAA_P_Ctrl),by = NULL)->lm_dev_rate_mean3
summary(lm_dev_rate_mean3,infer=c(TRUE,TRUE))


###############
### Weight     ###
###############

droplevels(subset(BCAA_data2,sex=="Female"))->BCAA_female
head(BCAA_female)

###### Analysis ######
head(BCAA_female)
lmer(log(Weight_per_female)~regime*treatment+ (1|experimenter)+(1| Pop)
+(1|treatment:Pop)+(1|block), data =BCAA_female)->Weight_per_female
anova(Weight_per_female)

###################
### Growth rate     ###
###################

droplevels(subset(BCAA_data2,sex=="Female"))->BCAA_female
head(BCAA_female)
BCAA_female$treatment<-factor(BCAA_female$treatment, levels = c("poor","BCAA","WHK"))

log(BCAA_female$Weight_per_female/0.005)/(BCAA_female$day_collect-5)->
BCAA_female$growth_exact_day

###### Analysis ######
head(BCAA_female)
lmer(growth_exact_day~regime*treatment+ (1|experimenter)+(1| Pop) +(1|treatment:Pop)+(1|block),
data =BCAA_female)->Growth_exact_lm
anova(Growth_exact_lm)
emmeans(Growth_exact_lm,"food")->Growth_exact_lm0
pairs(Growth_exact_lm0)
```

```
####################################################################################
##############################                               #####################################
############################## 15N enrichment #####################################
##############################                              ######################################
####################################################################################

read.table("Raw_data_15N enrichment 2 blocks.txt",h=T)->N_high_high
head(N_high_high)
N_high_high$block<-factor(N_high_high$block)
droplevels(subset(N_high_high,sample_type=="fly"))->N_high_high_fly

lmer(d15_fly_minus_diet~regime*stage*food+(1|population)+(1|population:stage)+(1|population:food
)+(1|population:food:stage)+(1|block),data=N_high_high_fly,REML=F)->lm1_all_minus_ML
anova(lm1_all_minus_ML)
```
