# Supplementary figures and images for: Experimental evolution of metabolism under nutrient restriction: enhanced amino acid catabolism and a key role of branched-chain amino acids
Source: Evol Lett. 2023 May 18;7(4):273–84. doi: 10.1093/evlett/qrad018 (PMC10355184; doi:10.1093/evlett/qrad018)

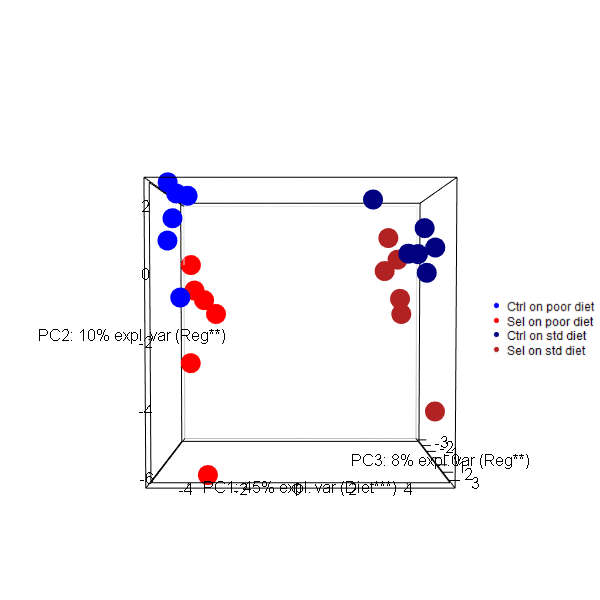

Supplement: qrad018_suppl_Supplementary_Figure_S1 [file qrad018_suppl_supplementary_figure_s1.gif]
